# Protocol of a randomized controlled trial of an erythropoietin stimulating agent decision aid for anemia treatment in kidney disease

**DOI:** 10.1186/s12882-016-0301-z

**Published:** 2016-07-18

**Authors:** Lauren B. Beach, Marcus Wild, Gowri Ramachandran, H. Omer Ikizler, Kerri L. Cavanaugh

**Affiliations:** Division of Nephrology & Hypertension, Vanderbilt University Medical Center, 1161 21st Avenue South, S-3223 MCN, Nashville, TN 37232 USA; Vanderbilt Center for Kidney Disease, Nashville, TN USA; University of Toledo College of Medicine, Toledo, OH USA; University of Vermont College of Medicine, Burlington, VT USA

**Keywords:** Shared decision making, Decision aid, IPDAS, Anemia, Chronic kidney disease, Erythropoietin stimulating agent, Dialysis, Patient provider communication, End stage renal disease

## Abstract

**Background:**

Erythropoiesis-stimulating agents (ESAs) are commonly used for the treatment of anemia due to chronic kidney disease (CKD) and end stage renal disease (ESRD). Patients often lack an understanding of the potential risks and benefits of ESAs, despite government mandated education on this topic. Decision aids are tools commonly used to discuss important information in health care settings. To address this knowledge gap, we designed this study to evaluate the effectiveness of a novel ESA decision aid at promoting informed shared decision making (ISDM) between patients and providers related to ESA use for CKD- and ESRD-related anemia.

**Methods:**

Using the principles of informed shared decision making theory, we designed and piloted an ESA decision aid intended to increase CKD and ESRD patient understanding of the potential risks and benefits of ESAs. Informed by the findings during development, the ESA decision aid was modified and finalized for testing. We will perform a randomized clinical trial to assess if administration of the ESA decision aid improves patient understanding of the risks and benefits of ESA use compared to control patients receiving standard care. Participants with either CKD or ESRD and who are receiving ESAs will be eligible for participation. The primary outcome is patients’ score on the Patient Anemia Knowledge in Kidney Disease (PAKKD) survey assessed at enrollment and 3 months after. Secondary outcomes include decisional conflict related to ESAs, and patient satisfaction with provider communication.

**Discussion:**

The Anemia Risk Communication for patients with Kidney Disease (ARC-KD) study will assess the effectiveness of a novel ESA decision aid to improve patient understanding of ESA use to manage CKD- and ESRD-related anemia. This decision aid is the first resource targeted to improve patient understanding of anemia management in the kidney health context. With the increasing options available for anemia management, this will serve as an important foundation to evolve in the future to optimize anemia-related shared decision making.

**Trial registration:**

ClinicalTrials.gov, number NCT01992926. Registered 11/14/2013.

## Background

Anemia affects nearly 95 % of patients receiving dialysis and 15 % of those with chronic kidney disease (CKD) [1]. Erythropoiesis-stimulating agents (ESAs) have been used for the past two decades to treat the complications of anemia in patients with kidney disease. The inability of ESAs to improve patient-reported outcomes, such as fatigue, in clinical trials has cast doubt on the potential benefits of ESAs relative to their risks, which include a near doubling of risk for stroke [2]. In March 2010, the Food and Drug Administration (FDA) required the administration of medication guides to patients receiving ESAs to improve their understanding of the potential risks and benefits of ESA treatment [3]. Unfortunately, these basic tools may not adequately educate patients thoroughly about the risks and benefits of ESA therapy. Even patients with kidney disease who frequently visit their nephrologists have knowledge deficits about their disease and the role of the kidney in anemia [4]. This is likely in part because communication is difficult from both the patient and the physician perspectives, and this is amplified when discussing possible risks [5].

Informed shared decision making (ISDM) has emerged as a leading evidence- based theory that, when applied successfully, can improve patient-provider communications surrounding health risks [6]. Best-practice ISDM requires the provider to initiate conversations by introducing the concept of patient choice, describe the options of care available to patients, often by employing a decision aid, and help patients better define their preferences to make good health care decisions [7]. The Ottawa Decision Support Framework, an evidence-based set of guidelines used in the development and evaluation of over 30 empirically derived decision aids, also requires that the ISDM process be critically evaluated for quality and its impact on health outcomes [8, 9]. Decision aids developed by systematically applying ISDM theory can help to reinforce consistent application of ISDM principles during the health care decision making process [10].

In this study, we sought to develop and evaluate a decision aid for ESA therapy to be administered to ESRD and CKD patient populations. We hypothesized that the use of an ESA decision aid that follows the International Patient Decision Aid Standards (IPDAS) [11] and adheres to expert opinion for optimizing communication with low health literacy and numeracy patients [12] will increase patient knowledge of anemia in kidney disease, reduce patient uncertainty related to ESA use, and increase overall patient care satisfaction. We also hypothesize that these effects might be enhanced for vulnerable patient populations, who stand to benefit from these improvements the most [12, 13].

## Methods and study design

### Study design summary

**Fig. 1:**
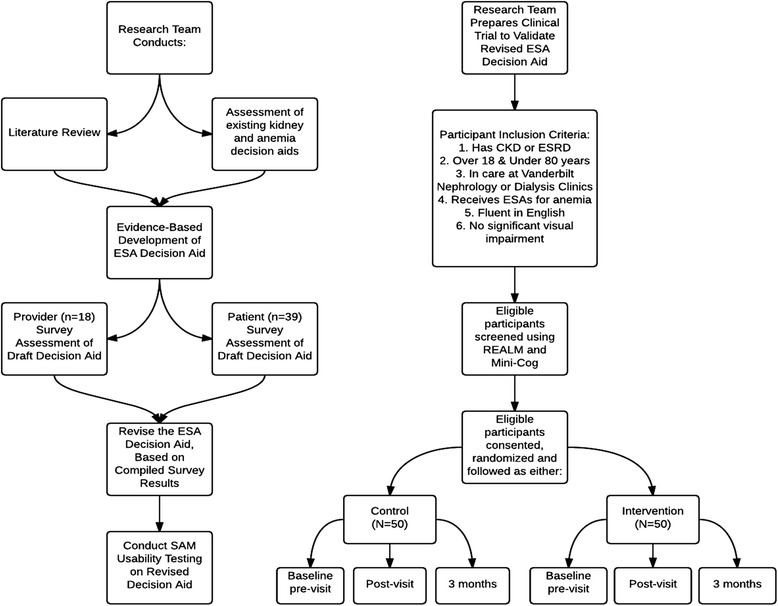
ARC-KD Study Planning Diagram. Phase 1 (*left side*). Development of the ESA decision aid. Phase 2 (*right side*) randomized clinical trial to evaluate effect of ESA decision aid

The Anemia Risk Communication for patients with Kidney Disease (ARC-KD) study is a two-phase study to design, test, and optimize a novel decision aid to improve ISDM and patient understanding regarding the use of ESAs for treatment of anemia in chronic kidney disease (Fig. 1). In Phase 1, we designed, revised, and usability-tested a one-page ISDM-informed ESA decision aid intervention. In Phase 2, we will conduct a randomized controlled trial to assess the effectiveness of the revised ESA decision aid at improving CKD and ESRD patient knowledge and decision making about anemia management using ESAs.

### Phase 1a: development of the ESA decision aid

To prepare for Phase I implementation, we conducted a literature review to support the development of the ESA decision aid. We focused our search on papers that described theories of ISDM in medical practice using the Ottawa Decision Support Framework. We also reviewed examples of high quality decision aids in the Decision Aid Library Inventory (DALI) available online via the Ottawa Hospital Research Institute [14]. This search revealed limited research and resources describing kidney-specific decision aids and none describing anemia management decision aids, kidney health related or otherwise [13]. Importantly, however, we did find evidence that the application of ISDM principles to patient-provider communications can improve health knowledge and outcomes in CKD and ESRD patient populations, including in patient sub-populations with low health literacy and numeracy [12].

This search led us to consider the IPDAS quality domains as guiding principles when developing the ESA decision aid [11]. These domains define high quality decision aids as those that [1] [involve a] systematic development process [2] provide information about options [3] present probabilities [4] clarify and express values [5] use patient stories [6] guide or coach [patients and providers] in deliberation and communication [7] disclose conflicts of interest [8] deliver patient aids on the internet [9] balance presentation of options [10] use plain language [11] base information on up to date scientific evidence and [12] establish effectiveness [11]. The minimum quality domains and standards necessary to certify high-quality, ISDM-informed decision aids under the IPDAS framework are still undergoing consideration and are not yet defined [15].

**Fig. 2:**
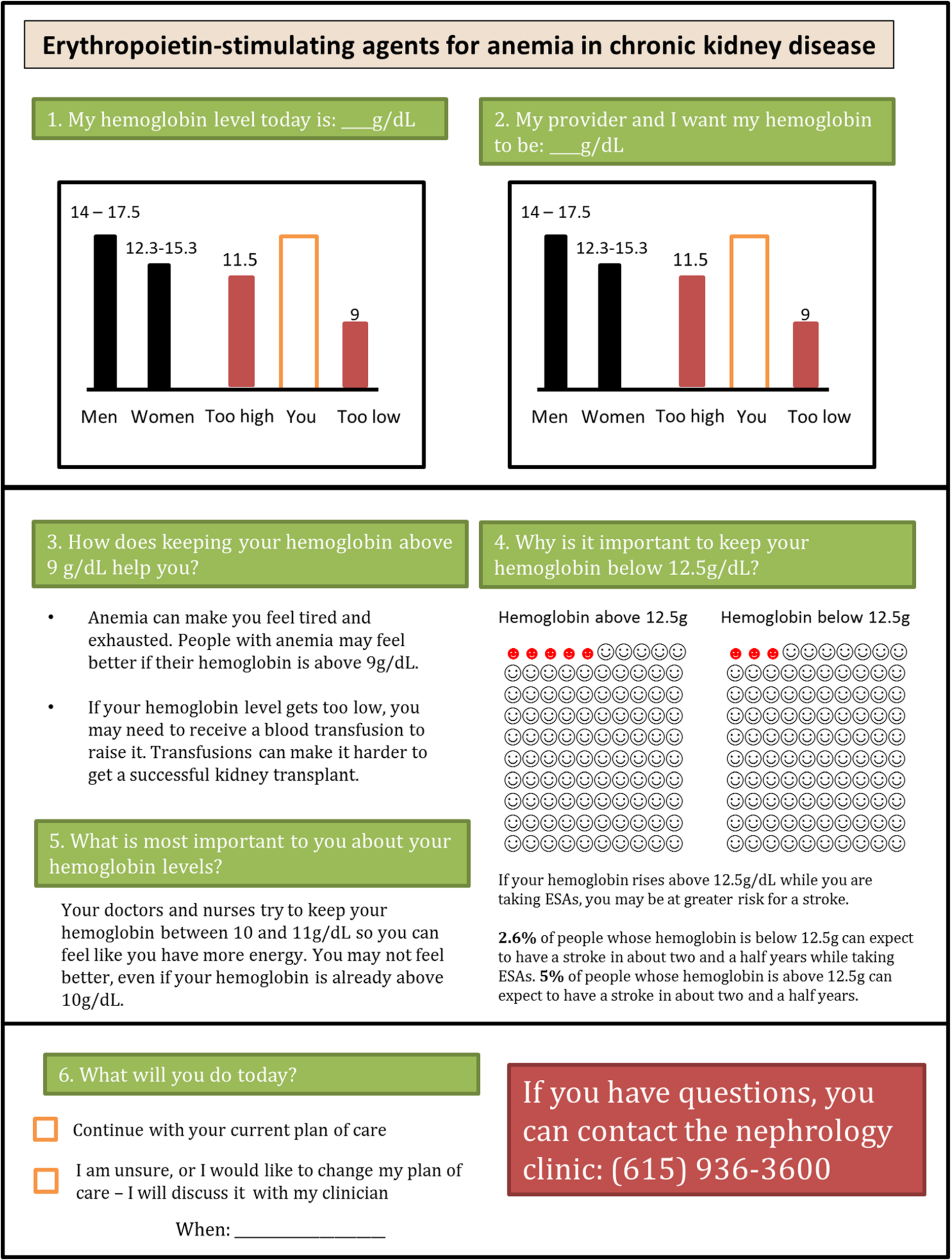
Draft ESA Decision Aid. The draft ESA decision aid contains 6 sections organized to be delivered to patients from *right* to *left*, then *top* to *bottom*. The “Men” and “Women” bars in Sections 1 and 2 represent the hemoglobin levels of individuals without kidney disease, while the “*too high*” and “*too low*” bars represent the high and low ranges of hemoglobin levels for individuals with kidney disease, respectively. Section 3 includes information to educate patients about the impact of anemia on patient health and wellness. Section 4 presents the potential health risks of using ESAs to manage anemia, while Section 5 presents the potential benefits. Section 6 contains checkboxes to assist patients with planning their future ESA use

The resulting draft ESA decision aid (Fig. 2) included 6 sections numbered to indicate the order in which the ESA decision aid should be administered. On its face, the systematically developed (IPDAS criterion 1) draft ESA decision aid contained sections that required the patient and provider to set shared goals (IPDAS criterion 6) (Section 1); encouraged the patient to engage interactively with the aid to facilitate individual consideration of acceptable treatment risk vs. quality of life benefits (IPDAS criteria 4) (Sections 2 and 6); helped the patient learn basic facts about anemia (IPDAS criteria 2, 9 and 11) (Sections 1–3); helped the patient understand the probability-based risks and benefits of ESAs to treat anemia and the resulting potential impacts on quality of life (IPDAS criterion 3) (Sections 4 and 5); and allotted space for the patient to make a care related decision regarding whether or not to use ESAs (when trial data is evaluated, will contribute toward achieving IPDAS criterion 12) (Section 6). Additionally, the ESA decision aid used plain language and was drafted at a seventh grade reading level to facilitate comprehension of the aid by all patients, including those with low health literacy (IPDAS criteria 10) [12]. In total, the draft ESA decision aid met 9 of the 12 quality domains from the 2006 IPDAS decision aid checklist.

The IPDAS domains not addressed by the draft decision aid were “5. using patient stories”, “7. disclosing conflicts of interest” and “8. delivering patient aids on the internet.” We chose not to include an individual patient story due to concerns that variability in the degree individual patients related to the patient in the story could bias patient decision-aid assisted decision-making results [11, 16]. Though the aid does not state it, no conflict of interest exists for any person who developed the decision aid. Lastly, because our trial planned to deliver the decision aid in person for research purposes, it was not made available online.

To further evaluate the quality of the draft ESA decision aid, we engaged patients and providers to review the usefulness and accessibility of the tool, in accordance with recent decision aid best practices [10]. A convenience sample of 18 nephrology medical providers and 39 patients with kidney disease at Vanderbilt University Medical Center provided feedback on the draft ESA decision aid. Patients providing feedback had a CKD or ESRD diagnosis, were at least 18 years of age, were receiving ESAs for anemia, spoke English, and had no significant visual impairment. No compensation was given to participants.

Patients and providers independently reviewed printed copies of the ESA decision aid and completed a 17-question survey to provide feedback about the design and usefulness of the ESA decision aid at promoting informed shared decision making. Results from the surveys were compiled and used to revise the ESA decision aid.

### Phase 1b: revision of the ESA decision aid

**Fig. 3:**
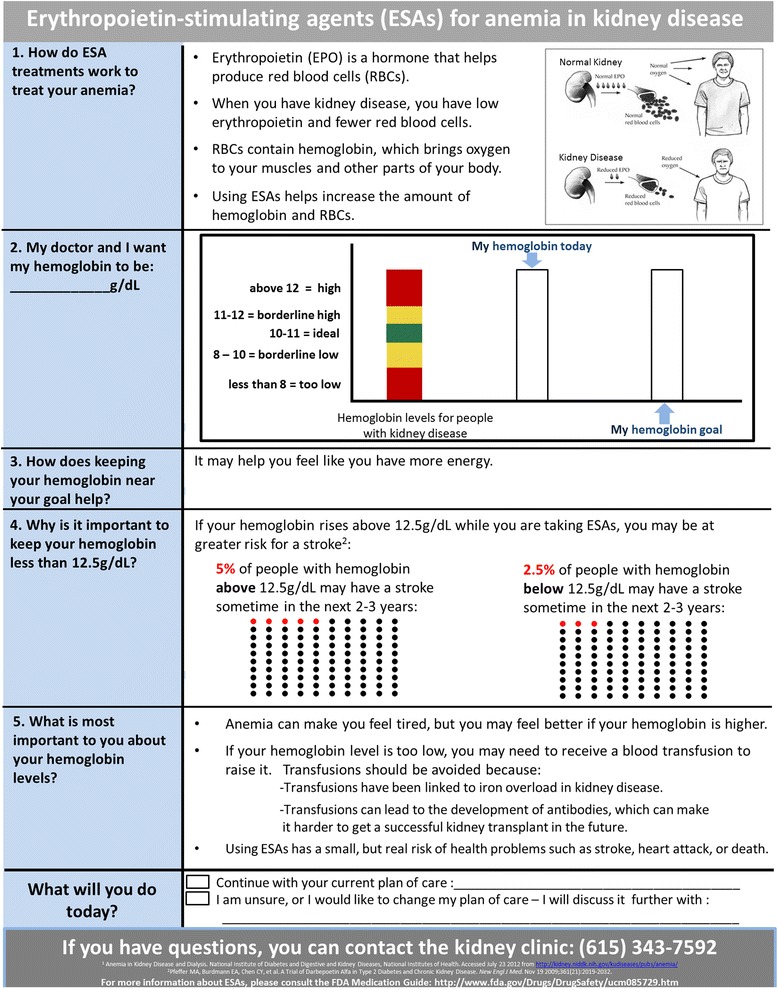
Revised ESA Decision Aid. The revised ESA decision aid contains 6 sections organized to be delivered to patients from *top* to *bottom*. Section 1 explains the mechanism for how and why ESAs are used to treat anemia in kidney disease patients. Section 2 raises patient awareness of their current hemoglobin level and encourages them to set a new goal hemoglobin level with their providers. Section 3 presents the benefits of ESAs, while Section 4 presents the risks. Section 5 contains additional information to help patients understand the impact of their decision to use ESAs on downstream treatment options related to broader kidney health outcomes. Section 6 includes checkboxes to assist patients with planning their future ESA use

Review and analysis of the survey feedback showed approximately half of patients and providers alike felt significant revisions to Sections 1, 2 and 4 of the draft decision aid were warranted. In Section 1 (presentation of healthy hemoglobin levels), patients described the inclusion of hemoglobin values for “normal” patients without kidney disease as stigmatizing. Generally, patients reported they found the graphs in Sections 1 and 2 (presentation of target hemoglobin levels for patients with kidney disease) to be more confusing than the informational sections of the decision aid. Likewise, providers predicted that such confusion about graphical interpretation may occur among patients. In response to this feedback, we revised Sections 1 and 2 of the decision aid to only include graphs containing pictorial representations of hemoglobin levels observed in CKD and ESRD patients (Figs. 2 and 3).

Additionally, both patients and providers expressed confusion about the use of smile face icons in Section 4 (pictorial representation of stroke risk due to ESA use) to represent symbolically the probability of adverse events due to ESA use. Patients reported these symbols were a poor choice to symbolize risk, because they associated smile faces with positive rather than with adverse health outcomes. In response, the revised ESA decision aid included solid dots in the pictograph (Fig. 3).

### Phase 1c: usability testing of the ESA decision aid

After revision, the ESA decision aid was evaluated for usability with the Suitability Assessment of Materials (SAM) survey [17]. SAM scores of 70–100 are superior, scores of 40–69 are adequate, while scores less than 40 are inadequate. Previous evaluation of 69 kidney disease patient education materials found a mean overall usability score of 58 % [17]. The ESA decision aid SAM score for the revised ESA decision aid was 89 %, demonstrating a superior mean SAM usability score. The next phase of this study will test this version of the decision aid informed by both provider and patient stakeholders.

### Phase 2: randomized controlled trial evaluating the use of an ESA decision aid to improve patient anemia knowledge

To evaluate the ESA decision aid in practice, we will conduct a randomized controlled trial of 100 patients. The trial will utilize validated survey tools and measures to determine if the primary and secondary outcomes of increased patient knowledge and patient satisfaction with ESA use decision have been met, respectively (Table 1).

**Table 1:** Study outcomes, measures, and patient characteristics

No.	Outcomes, Measures & Characteristics	Data Source by Outcome	Timeline and Frequency		
			Baseline Pre-Visit	Post-Visit	Three Months
1	Primary Outcome	*Patient Knowledge*			
	Anemia Knowledge	Patient Anemia Knowledge in Kidney Disease (PAKKD) [19]	X	X	X
2	Secondary Outcomes	*Patient Satisfaction*			
	Provider Communication Satisfaction	Satisfaction with Provider Communications (CAT) [20]		X	
	Health Decision Certainty	Decisional Conflict Scale (DCS) [21]		X	
	Perceived Communication Efficacy	Perceived Efficacy in Patient-Physician Interactions (PEPPI) [22]		X	X
3	Subject Characteristics	*Potential* *Co-Variables*/*Modifiers*			
	Patient Demographic Information	Demographic Form	X		X
	Patient Numeracy Assessments	Schwartz Risk Numeracy Test [27]	X		
	Anemia Symptoms	Functional Assessment of Chronic Illness Therapy-Fatigue (FACIT-F) [28]	X		X
	Medical Outcomes	Patient Electronic Medical Record Hemoglobin Lab Values	X		X

Following informed consent, ARC-KD study participants will complete the indicated survey metrics during at least one of three possible time points: enrollment (Baseline Pre-Visit), up to one week after the baseline enrollment visit (Post-Visit) and three months after enrollment (Three Months). Study staff will obtain hemoglobin lab values from each participant’s electronic health care record at baseline and three months.

#### Target population and eligibility criteria

Planned recruitment criteria for study participants include diagnosis of CKD or ESRD, current ESA therapy, age between 18 and 80, and English fluency. Patients will be excluded if they have significant cognitive impairment or visual impairment. Cognitive impairment will be determined by asking patients to complete the Mental Status Assessment of Older Adults, or Mini-Cog [18]. Participants will be excluded if they do not speak English, due to a lack of available staff to translate.

#### Recruitment of participants

Participants will be recruited from the in-center and home dialysis clinics at Vanderbilt University Medical Center, as well as the outpatient nephrology clinic at the Vanderbilt Clinic. Potential participants will be identified by an examination of their electronic medical record. Patients will be actively recruited by a study coordinator who will approach eligible individuals, explain the content of the study, and answer any questions the individual may have. Written informed consent will be obtained from all participants. Reminder phone calls and appointment cards are given to patients at all clinics participating in the study to increase patient appointment attendance. These methods will also be used to increase retention of study participants, who will receive the intervention or control usual care during a pre-scheduled patient appointment.

#### Randomization

Using blind and secure allocation, participants will be randomly assigned to one of two intervention arms: 1.) the usual care control group, or 2.) the ESA decision aid intervention group. Randomization will not be blocked by site, due to recruitment from within one large nephrology practice. The allocation sequence will be computer-generated. Participants will be recruited and assigned to intervention groups by the study coordinator, who will be blind to the allocation sequence. After assignment trial participants and care providers not involved in the study will not be blinded to the assignment.

#### Usual care group

Participants who are randomly assigned to the control group will receive standard care from their existing nephrologists, nurses, and clinical staff. As all of the participants in the study will be receiving ESA therapy, this care may include a description of the purpose of the medication by the staff member administering the injection and counseling about its purpose and potential risks, but without use of the ESA decision aid.

#### Intervention group

Participants randomly assigned to the intervention group will receive a paper copy of the ESA decision aid (Fig. 3). This decision aid will be administered by and discussed with a nephrology nurse practitioner, who will explain the purpose and function of ESA’s, the goal hemoglobin levels for those receiving ESAs, the positive and negative effects of the medication, and additional resources available to patients. In order to maintain consistency across the clinics, the same nurse practitioner will administer the decision aid tool to all intervention participants across the dialysis clinics. Similarly, a nurse in the CKD clinic that is assigned to anemia management will be responsible for administering the ESA decision aid. Each of these clinical personnel will be trained and oriented to the content and recommended use of the decision aid.

### Data collection, follow-Up, and outcomes

#### Measures assessed

All participants will be assessed using in-person questionnaires at baseline before or after their study visit and at 3 months after enrollment, as described in Table 1. Changes in patient scores that occur between baseline pre-visit and after the administration of the ESA decision aid will be compiled and analyzed. Additionally, all patients’ medical records will be abstracted for relevant comorbidities, lab values, medications, and vital signs at both baseline and 3 month study visits. In this minimal risk study monitoring for any reported associated adverse events such as anxiety with the educational intervention will be performed throughout its duration by the PI and study team. All available efforts will be made to blind outcome survey assessors from group assignment.

#### Data management, privacy and monitoring

Data will be entered into a secure online database by the study coordinator. To protect patient confidentiality, study-specific identification numbers will be used in the study database in lieu of participant identifying information. Only the PI and the study coordinator will have access to the study identification/patient identification link log, which will be destroyed after study completion. The PI will monitor data quality and data queries will be resolved by the study coordinator. Due to the low risk profile of the study, ARC-KD will not convene a data monitoring committee.

### Statistical considerations

#### Data analysis

The randomly assigned intervention or control group will be the primary independent variable for intention to treat analysis. Multiple imputations will be used to handle missing data. Evaluation of the primary outcome of patient anemia knowledge will be determined by comparing intervention and control group Patient Anemia Knowledge in Kidney Disease (PAKKD) [19] survey scores between baseline pre-visit and post-visit as well as baseline pre-visit and 3 months post-intervention for both study arms. Evaluation of the secondary analyses of provider communication satisfaction, health decisional certainty and perceived self-efficacy in patient-provider communications and patient kidney care will be similarly measured and analyzed using the Communication Assessment Tool (CAT) [20], Decisional Conflict Scale (DCS) [21] and Perceived Efficacy in Patient-Physician Interactions (PEPPI) [22] surveys, respectively. Baseline pre-visit variables will be compared between the study arms to assess the successfulness of randomization including gender, age, education and race, using median and interquartile ranges for continuous variables and frequencies and proportion for categorical variables to ensure homogeneity. Chi-squared tests for categorical variables and Wilcoxon-rank sum test for continuous variables will be used to assess the differences in baseline data between intervention and control. We will use linear regression models to compare the change in the PAKKD anemia knowledge scores from baseline to post-visit and baseline to 3 months post intervention between the study arms. Baseline pre-visit values of the outcome variable will be included as a model covariate in linear regression analysis to compare changes from baseline pre-visit. Lastly, planned exploratory analyses include performing logistic regression analysis to assess association between the likelihood of achieving or maintaining hemoglobin targets.

#### Sample size and power

We are aware of no other previous studies examining the effectiveness of a shared decision-making intervention designed for the use of ESAs in CKD or ESRD. Based upon previous experience of using a brief educational worksheet to improve general kidney knowledge [23], we estimate a possible doubling or tripling improvement in the odds of improved kidney health knowledge. For the primary outcome of the PAKKD anemia knowledge score (range: 0–100 %), preliminary data have showed a mean (standard deviation) improvement of 59 % (20 %) [19]. Using a *t*-test for independent samples, the calculated minimum detectable difference is 11 % (59 % in control, 70 % in intervention), showing 19 % relative improvement in anemia knowledge score with 80 % power at a 2-sided significance level. For the purposes of this study, the proposed sample size of 100 participants should be adequate and informative.

## Discussion

It has been acknowledged from multiple levels of care and governance that communication of risk and proper use for ESAs in the treatment of anemia in CKD and ESRD is an area of concern that must be addressed [24]. We designed the Anemia Risk Communication for patients with Kidney Disease (ARC-KD) trial to create and evaluate the effectiveness of a novel ESA decision aid to assist CKD and ESRD patients and providers in deciding whether or not to use ESAs to manage anemia related to underlying kidney disease. Well-designed decision aids have been shown in randomized controlled trials to improve health knowledge, lower decisional conflict, and promote accurate risk perceptions for patients living with a variety of complex health conditions [25]. Decision aids well-designed to address barriers to ISDM for vulnerable patient populations, such as patients with limited health literacy, have been shown to improve patient knowledge. In diabetics, such aids have even been implicated in achieving tighter glycemic control [26]. After study completion we will make the decision aid available via the internet for review by the public and use by health professionals.

Our study will make advances in a clinically important, but understudied, area of kidney disease care. It will also allow us to assess whether this ESA decision aid is associated with higher patient knowledge of anemia, as well as their satisfaction and certainty regarding their decision to use ESAs. By using the ISDM approach to improving patient understanding of the risks associated with ESA use in CKD, we hope to mitigate barriers to effective patient-provider communication such as low health literacy, and develop a resource for providers to use cooperatively with their patients. Patient feedback on the utility of the revised ESA decision aid indicated kidney disease patients who have only recently started receiving anemia treatment may receive the maximum benefit from the implementation of the decision aid tool. Interestingly, patient feedback also suggested the tool could be useful in explaining the impact of anemia to family members. Importantly, as the pharmacologic options for treatment of anemia in kidney disease expand in the future, a kidney-specific anemia therapy decision aid may become a required component of optimized patient-centered care.

## Abbreviations

ARC-KD, the anemia risk communication for patients with kidney disease study; CAT, communication assessment tool; CKD, chronic kidney disease; DALI, decision aid library inventory; DCS, decisional conflict scale; ESA, erythropoiesis-stimulating agents; ESRD, end stage renal disease; FDA, food and drug administration; IPDAS, international patient decision aid standards; ISDM, informed shared decision making; PAKKD, patient anemia knowledge in kidney disease; PEPPI, perceived efficacy in patient-physician interactions
